# Antibacterial Efficiency of *Tanacetum vulgare* Essential Oil against ESKAPE Pathogens and Synergisms with Antibiotics

**DOI:** 10.3390/antibiotics12111635

**Published:** 2023-11-17

**Authors:** Horațiu Roman, Adelina-Gabriela Niculescu, Veronica Lazăr, Mihaela Magdalena Mitache

**Affiliations:** 1 Interdisciplinary School of Doctoral Studies (ISDS), University of Bucharest, 050095 Bucharest, Romania; horatiu.roman@bio.unibuc.ro; 2 Research Institute of the University of Bucharest (ICUB), University of Bucharest, 050095 Bucharest, Romania; 3 Faculty of Biology, University of Bucharest, 050095 Bucharest, Romania; veronica.lazar@bio.unibuc.ro; 4 Department of Science and Engineering of Oxide Materials and Nanomaterials, National University of Science and Technology Politehnica Bucharest, 011061 Bucharest, Romania; 5 Department of General Medicine, Titu Maiorescu University, 040441 Bucharest, Romania; magdalena.mitache@prof.utm.ro

**Keywords:** natural products, *Tanacetum vulgare*, tansy extract, multidrug resistance, bioactive compounds, antibacterial activity, synergism with antibiotics

## Abstract

Medicinal plants with multiple targets of action have become one of the most promising solutions in the fight against multidrug-resistant (MDR) bacterial infections. *Tanacetum vulgare* (Tansy) is one of the medicinal plants with antibacterial qualities that deserve to be studied. Thus, this research takes a closer look at tansy extract’s composition and antibacterial properties, aiming to highlight its potential against clinically relevant bacterial strains. In this respect, the antibacterial test was performed against several drug-resistant pathogenic strains, and we correlated them with the main isolated compounds, demonstrating the therapeutic properties of the extract. The essential oil was extracted via hydrodistillation, and its composition was characterized via gas chromatography. The main isolated compounds known for their antibacterial effects were α-Thujone, β-Thujone, Eucalyptol, Sabinene, Chrysanthenon, Camphor, Linalool oxide acetate, *cis*-Carveol, *trans*-Carveyl acetate, and Germacrene. The evaluation of the antibacterial activity was carried out using the Kirby–Bauer and binary microdilution methods on Gram-positive and Gram-negative MDR strains belonging to the ESKAPE group (i.e., *Enterococcus faecium*, *Staphylococcus aureus*, *Klebsiella pneumoniae*, *Acinetobacter baumannii*, *Pseudomonas aeruginosa*, and *Enterobacter* spp.). Tansy essential oil showed MIC values ranging from 62.5 to 500 μg/mL against the tested strains. Synergistic activity with different classes of antibiotics (penicillins, cephalosporins, carbapenems, monobactams, aminoglycosides, and quinolones) has also been noted. The obtained results demonstrate that tansy essential oil represents a promising lead for developing new antimicrobials active against MDR alone or in combination with antibiotics.

## 1. Introduction

The therapy of infectious diseases is going through a major crisis due to the extent of the phenomenon of antibiotic resistance (AR), which has reached alarming proportions. The World Health Organization (WHO) list (27 February 2017) [1] of priority pathogens for research and development of new antibiotics comprises three priority levels: critical, high, and medium. The critical level includes multidrug-resistant pathogens (MDR—bacteria resistant to three or more classes of antibiotics) ESKAPE, Gram-negative pathogens, *A. baumannii* (carbapenem-resistant), *P. aeruginosa* (carbapenem-resistant), *K. pneumoniae* (resistant to third-generation cephalosporins), and Enterobacter spp. (resistant to third-generation cephalosporins). The high level includes ESKAPE Gram-positive pathogens, *Enterococcus faecium* (vancomycin-resistant), and *S. aureus* (intermediate methicillin-resistant and vancomycin-resistant). These pathogens are often isolated from clinical settings associated with life-threatening nosocomial infections [2].

The design of new (classes of) antibiotics is difficult to achieve. Although efforts are made to stop and reduce the phenomenon, the monitoring studies do not report the positive effects of these policies, as the incidence of multidrug and pandrug-resistant strains is continuously increasing [3]. New antibiotics that entered the market in recent decades are variations of pre-existent antibiotics discovered up to the 1980s [4]. The lack of progress in antibiotic development highlights the need to explore innovative approaches to treating bacterial infections.

The list of plants with curative effects against a wide variety of chronic and infectious diseases has expanded, especially in recent decades. Analytical methods have identified diverse compounds with therapeutic effects, especially against bacterial infectious diseases, in the context of the expansion of the phenomenon of antibiotic resistance [5,6]. In vitro tests have shown that some plant species that are used to treat non-infectious diseases (e.g., *Panax ginseng*, *Achillea* sp., *Cichorium intybus* L., *Cynara cardunculus* L., *Foeniculum vulgare* Mill., which regulate liver functions [7], or plants that regulate the nervous system, such as *Ginkgo biloba* L., *Panax ginseng* C.A. Mayer, *Scutellaria baicalensis* Georgi [8]) also have antibacterial activity [9,10,11,12,13,14]. More than a quarter of the medicines administered in industrialized countries are obtained directly or indirectly from plants. The antimicrobial action of plant extracts results from their complex chemical composition, the compounds acting synergistically and having multiple action targets. These preparations are generically referred to as “non-antibiotic” antibacterial agents [15] and were proven to be active against a large spectrum of bacterial and fungal strains [16].

The use of essential oils (EOs), resulting from the secondary metabolism of plants, has been known since Neolithic times when they were extracted via pressing. The Egyptians used to extract oils by infusing a plant into a fatty substance. By boiling, the flavor evaporates and is fixed in the fat [17]. The oldest medicine book, “Shennong’s Herbal”, dating back to 2700 BC, contains instructions for using 365 medicinal plants belonging to Chinese folklore. Hippocrates, the father of modern medicine, documented the medical benefits of fumigation with aromatic oils in treating plague. There are 12 different types of EOs mentioned in the Holy Bible. In 1990, in the book “L’Aromatherapie Exactement”, the medical properties of more than 270 EOs are described, representing a start for many studies [18].

The main chemical compounds found in the composition of EOs include terpenes, terpenoids, alkaloids, and phenolic compounds. EOs are obtained from raw vegetable material, steam distillation, or dry distillation. Yields vary greatly depending on plant parts’ pre-processing techniques and agronomic factors (climate, soil, oral harvest interval) [19,20]. In general, the increase in drying temperature is proportional to the decrease in the EO content [21]. Thus, drying plants at 30 °C leads to concentration losses of 16%, while drying at higher temperatures of 60 °C leads to 65% losses [22]. Therefore, the obtaining process is directly correlated with the quality of the EOs.

Tansy (*Tanacetum vulgare)* is an aromatic plant that combines the smell of *Achillea millefolium* with *Artemisia absintum*, *Piper nigrum*, *Salvia mirzayanii*, and *Eucalyptus* sp. due to some common aromatic compounds. Aromatic plants are rich in essential oils. The term “essential oil” was used by Paracelsus von Hohenheim, who called the effective component of a medicine “Quinta essential”. Conventional methods of obtaining essential oil include cold pressing, distillation, and solvent extraction. Supercritical and subcritical carbon dioxide (CO_2_) extraction methods are more advanced and used industrially. The most efficient method, both financially and in terms of working time, is via water vapor distillation, which we conducted in this work.

Although *Tanacetum vulgare* is a plant that has been studied a lot recently due to its multiple therapeutic properties, including anti-inflammatory, antioxidant, and antibacterial activities, it still presents novelties due to its chemical compounds that vary according to environmental conditions (e.g., soil pH, light exposure, humidity, pollution) [23,24,25]. According to the World Checklist of Selected Plant Families, Anthos, Flora Iberica, and other databases, it has been attributed to numerous names correlated with its action/benefits, such as the gate of heaven, herb of the Mother of God, herb of the air, herb of worms, triac plant, medicinal of St. Anastasia, moss herb, St. Mark’s weed, bitter weed, St. Teresa’s pen, frankincense, and mugwort. However, it has been destroyed by herbicide or incineration in recent years, being considered an invasive plant.

In Romania, Tansy has a wide distribution, including all geographical areas. The best harvesting period is considered to range from July to August. The plant used herein was selected from an area with abundant growth in the Sohodol Valley area, Gorj County, Romania.

This study aims to highlight the potential of the EOs extracted from locally collected *T. vulgare* to be used as a source of antibacterial formulations or compounds. In this respect, EOs have been extracted via hydrodistillation, their main chemical constituents have been identified via gas chromatography-mass spectrometry (GC-MS), and their antibacterial properties have been investigated against clinically relevant Gram-positive and Gram-negative MDR bacterial strains, demonstrating promising activity. Thus, this study seeks to emphasize the importance of local plants for developing antimicrobial formulations against ESKAPE pathogens.

## 2. Results

The essential oil yield was 0.43% (*w*/*w*) after 6 h of distillation. The refractive index was determined using an Abbe Zeiss refractometer, being 1.4756. The density measured with an ISOLAB pycnometer was 0.95783 g·mL^−1^.

**Figure 1:**
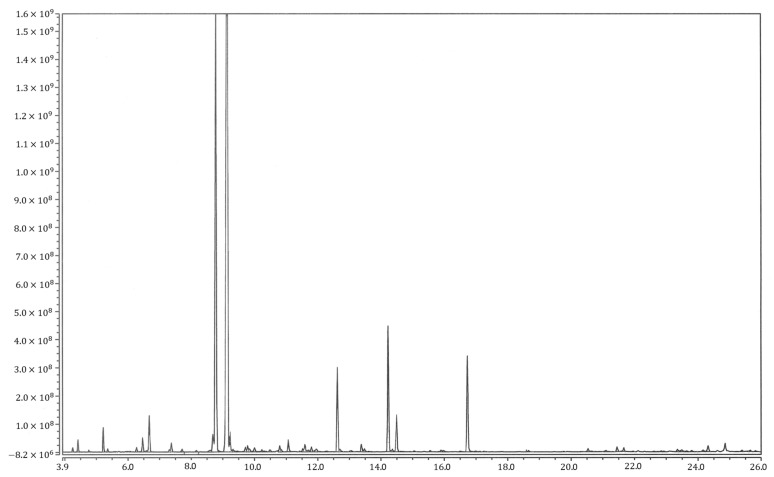
Chromatograph of EOs obtained from *T. vulgare*. The peaks indicate the concentration of the compounds.

Further, approximately 80 compounds were identified via GC-MS analysis (Figure 1) and summarized in Table 1 in the order of elution.

The chemical and active properties of Tansy depend on several factors (e.g., plant distribution, geographical location, and environmental factors) [26], which influence the yield and quality of the plant. Thus, we made a comparison between the main compounds obtained by us and those obtained and published in the specialized literature from different geographical areas: Poland, Serbia, and Canada. The working methods for obtaining the essential oil and identifying the compounds were similar, but the reports were different, suggesting the Tansy species’ variety. Table 2 summarizes these differences.

**Figure 2:**
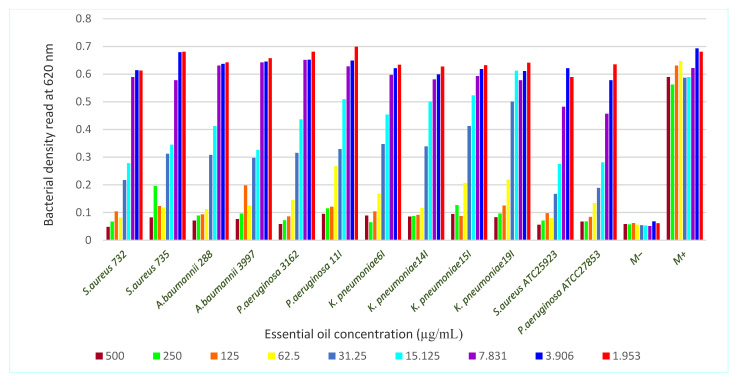
Graphic representation of the antibacterial activity of tansy EOs against the MDR strains. On the ordinate is written the value of the optical density of the cultures measured indirectly via optical density at 620 nm with a UV-vis spectrometer (Lambda25, PerkinElmer, Inc., Waltham, MA, USA), and on the abscissa is written the mass concentration of the Tansy extract (µg/mL). The best activity was recorded against the *S.aureus* strain isolated from wound secretions (31.25 μg/mL). M+ positive control (bacterial culture without extract addition) and M− (sterile culture medium). Experiments were performed in triplicate (*n* = 3), and results were expressed as means ± SD. Statistical analyses were performed using GraphPad Prism 7. If the probability of the difference was less than *p* < 0.05, it was deemed to be statistically significant.

In the quantitative assay of the antimicrobial activity of the tansy EOs, all tested strains proved to be very susceptible to the tested EOs in the mass concentration range of 62.5–500 μg/mL (Figure 2). The results obtained showed that Tansy had a remarkable antibacterial effect. Tansy essential oil inhibited the growth of almost all tested bacteria at a concentration lower than 61.5 µg/mL. A higher mass concentration (23.25 mg/mL) was needed to inhibit the growth of *P. aeruginosa* (125  µg/mL).

The *T. vulgare* extract had a different effect on the activity of the tested antibiotics, depending on the bacterial strain and the antibiotic. Table 3 summarizes the obtained results regarding the synergism of tansy EOs with the tested antibiotics. Each experiment was repeated three times, the diameter of the inhibition zone being the arithmetic mean of these values. The tolerance was ±0.5 mm.

As shown in Table 4, the Tansy EOs exhibited a synergic effect on all tested antibiotics in the case of *A. baumannii* and *K. pneumoniae* strains and on at least one antibiotic from each of the tested class in the case of *P. aeruginosa* strains (Table 3).

Plant extracts have multiple mechanisms of action on microorganisms. To better understand their mechanisms of bactericidal or bacteriostatic action, we evaluated the influence of extracts by associating them with different classes of antibiotics: aminoglycosides, ꞵ-lactams, and quinolones. In particular, the serial microdilution method was used to quantify the synergistic effect of tansy extract with antibiotics. Antibiotics, in combination with the Tansy extract, demonstrated enhanced antibacterial activities (Table 5).

As observed in Table 5, regarding the synergism of the *T vulgare* extract with the antibiotic, the plant extract had a different effect on the activity of the associated antibiotic and also depending on the bacterial strain. The zones of inhibition had values between 14 and 30 mm. Each experiment was repeated three times, the diameter of the inhibition zone being the arithmetic mean of these values. The tolerance was ±0.5 mm.

The synergism of the plant extract with the antibiotic was evident for all classes of antibiotics inhibiting bacterial growth. The potentiation of antibacterial activity of antibiotics in combination with plant extracts can be correlated with the synergism of the specific mechanisms of action of antibiotics and extracts on multidrug-resistant bacteria. Specifically, in combination with the extract, antibiotics have regained their properties as bacterial inhibitors. Moreover, the synergistic action of Tansy extract with antibiotics on MDR strains suggests the complexity of the action of the compounds resulting from secondary metabolism.

## 3. Discussion

The list of plants with curative effects against a wide variety of organic and infectious diseases has expanded, especially in recent decades, as analytical methods have identified compounds with therapeutic effects, especially against bacterial infectious diseases, in the context of the expansion of the phenomenon of antibiotic resistance. In vitro tests have shown that some plant species used to treat non-infectious diseases have antibacterial activity. More than a quarter of the medicines administered in industrialized countries are obtained directly or indirectly from plants [15].

The antimicrobial action of plant extracts results from their complex chemical composition, the compounds acting synergistically and having multiple action targets. These preparations are generically referred to as “non-antibiotic antibacterial agents” [15] and were proven to be active against a large spectrum of bacterial and fungal strains [16].

In this context, the main objective of this study was to evaluate the antibacterial effect of Tansy EO as well as its potential synergy with antibiotics. In this regard, the first step assumed the identification of the bioactive compounds of *T. vulgare* extract, comparing them with those reported by other authors. The variation in the concentration of the compounds is correlated with the site from which the plant was harvested [23]. In more detail, the essential oil composition indicates plant adaptation to habitat conditions and stress conditions: drought, radiation, high temperature, heavy metal content, and predators [28]. Thus, essential oils change according to environmental conditions [29].

The biological activity of Tansy extract is related to the total terpene content of the essential oil. As depicted in Table 2, Radulović et al. [27] identified the presence of four different chemotypes of these species in Serbia (thujone, *trans*-chrysanthenyl acetate, chrysanthenyl, and camphoric chemotype) depending on the geographical areas. The chemotype of the species studied by Coté et al. [24] collected from Chicoutimi, Quebec City, QC, Canada, is camphoric (30.4%). The chemotype of the species studied by Nurzyńska-Wierdak et al. [23], from eastern Poland (Wohyń Commune, Radzyń Poviat, Lubelskie Voivodeship) is *trans*-chrysanthenyl (76.09%). The chemotype of our plant was *trans*-Carvyl acetate (34.44%).

The compounds identified in this study had approximately the same concentrations as those reported in the cited literature, with some differences that could influence the higher antibacterial activity. Thus, Eucalyptol, *trans*-β-Ocimene, *cis*-Pinocamphone, *cis*-Carveol, (z)-Linalool oxide acetate (pyranoid), α-Fenchene, *trans*-Carvyl acetate, β-Copaene, Cedrol, and Junelol were not identified in these studies [23,24,27]. Therefore, this article completes the knowledge regarding *T. vulgare* EO, identifying and quantifying a series of previously disregarded compounds.

Eucalyptol is an important monoterpene in this study, found in a proportion of 2.47%, being widely used in the pharmaceutical industry, having anti-inflammatory properties, and killing leukemic cells in vitro [30]. Antibacterial activity has been reported in several studies [31,32], the main mechanism of action being the penetration of the bacterial cell membrane and its lysis [33]. Trans-β-Ocimene is a terpene that establishes tri-trophic interactions [34], which was identified in a proportion of 0.07%. In addition to its quality as a food additive, it has several therapeutic properties, including anti-inflammatory, antifungal, and antiviral effects [35,36]. *Cis*-Pinocamphone is a monoterpenoid found especially in *Xylopia aromatica* and *Aloysia gratissima*. *Cis*-Carveol is a monoterpene that has the ability to cause the death of the bacterial cell via the disintegration mechanism of the cell membrane [37]. α-Fenchene is a monoterpene with a camphor-like odor. The presence of this compound in plants has a role in increasing antioxidant activity [38]. The greatest spread was found in Artemisia sp., hence the similarity of the smell of the two plants [39].

*Trans*-Carvyl acetate is a terpene ester with a mint flavor with a role in increasing antibacterial activity [40]. β–Copaene is a sesquiterpene used as a food additive and flavor due to its antioxidant properties [41]. Cedrol is a sesquiterpene found in conifers used in traditional medicine, especially due to its antimicrobial properties [42]. Juneol is a sesquiterpene identified in high concentrations in *Bursera graveolens*, a tree that belongs to the same family (Burseraceae) as frankincense and myrrh with several therapeutic properties, including antibacterial [43].

Concerning identified concentrations, the most abundant compounds were carvyl acetate (34.44%) and β-thujone (30.26%). Carvyl acetate is a flavonoid predominantly found in *Mentha spicata* L. with strong inhibitory potential against Gram-positive and Gram-negative bacteria and pathogenic fungi [44]. The quality of *Mentha longifolia* (L) EOs is proportional to the carveol content [45]. On the other hand, α-Thujone and β-thujone are the two stereoisomeric forms by which thujone is represented in the ketone group of bicyclic monoterpenes [46].

Plants that have significant thujone content, such as *Artemisia* sp., *Achillea* sp., *Thuja* sp., and *Salvia* sp., are often used to flavor foods and beverages. Absinthe, for example, is a well-known alcoholic drink called absinthe after the name of the plant used due to the flavor obtained by adding the extract of *Artemisia absinthium* L. Thujone has long been thought to cause adverse psychoactive effects. However, recent research has made convincing arguments for correctly identifying substances with possible side effects in absinthe, such as ethanol and other compounds used in the adulteration process [47]. In addition, α-Thujone is the active ingredient of wormwood oil and other drugs and has been reported to have antinociceptive, insecticidal, and anthelmintic activity [48]. The toxicity of thujone has been studied extensively, neurotoxicity being the main toxic outcome [49]. Regarding the degree of toxicity of the two forms of thujone, α-Thujone has a higher toxicity than β-thujone. As thujone is found in many medicinal plants to eliminate the supposed negative effect of this compound, HMPC (Committee on Herbal Medicinal Products) and EMA (European Medicines Agency) were able to set the maximum daily dose limit for thujone, which was set between 3.5 and 5.0 mg/person [50].

Thujone formation is not a universal phenomenon in plants. The indirect precursor of thujone, sabinene, is one of the most widespread monoterpene compounds in EOs. Various external and internal factors influence the accumulation of thujone. The isomerization of thujones can be explained by the presence of intermediates such as sabinol, sabinone, and related compounds in EOs [51]. The thujone pathway exists in many sabinene-containing species, but the expression of the corresponding genes is repressed due to different metabolic interactions, leading to the lack of thujone (for example, plants of the *Lamiaceae* family) [52].

It has been shown that α, β-Thujone (70% of α-thujone and 10% of β-thujone) also have anti-cancer effects against placental choriocarcinoma cells by inducing apoptosis via the mitochondrial-mediated intrinsic pathway [53]. A study by Pudełek et al. [54] highlighted the properties of α-Thujone as a potent attenuator on the proliferation and viability of glioblastoma multiforme cells, as this compound displayed anti-invasive and pro-apoptotic effects on glioblastoma multiforme cells.

Further, the obtained extract was tested on Gram-positive and Gram-negative bacterial strains that have been selected to exhibit multiple drug resistance to current antibiotics, i.e., beta-lactams, aminoglycosides, and quinolones. In order to evaluate the antibacterial effects and the potential synergism with antibiotics, qualitative and quantitative assays have been performed. *T. vulgare* extract was observed to work together with antibiotics, achieving more significant antimicrobial effects. The combination of conventional antibiotics, against which bacterial strains have been resistant, with plant extracts can restore the effectiveness of treatment due to the synergistic antibiotic-extract effect [55].

The microorganisms analyzed were strains with multiple antibiotic resistance and reference strains. To evaluate the effects of the association of plant extracts with antibiotics, we determined the MIC values of plant extracts as a reference point for defining the interactions with antibiotics. The association of antibiotics with volatile oil extract from *T vulgare* has been investigated for possible synergistic interactions. Using the “chessboard” method, the synergism is manifested by the increased sensitivity of the microorganism in the simultaneous presence of both antimicrobial agents, which is reflected by changes in the values of the zones of inhibition [56]. This can be explained by the ability of Tansy extract to enhance bacterial cell membrane permeability [57], thus allowing higher amounts of antibiotics to enter pathogenic cells and consequentially destroy them.

The antimicrobial action of plant extracts is the result of all chemical compounds that act synergistically, having multiple targets of action. The minimum inhibitory concentrations of oil Tansy extract for the bacterial strains tested varied in the range (62.5 ÷ 500) μg/mL. In the quantitative assay, the most susceptible to the tested EOs were the *S. aureus* strains (for which the MIC was 125 µg/mL for *S. aureus*_732_ and *S. aureus*_735_ and 62.5 µg/mL for *S. aureus*_ATC25923_ compared to the results obtained by Coté et al. [24] for *S. aureus*_ATC25923_, of 59 µg/mL). For Gram-negative bacteria, the MIC values were approximately the same. Further, for *P. aeruginosa*_11I_, it was 125 µg/mL; for *P. aeruginosa*_3162_, it was 250 µg/mL; and for *P.aeruginosa*_ATCC27853_, it was 62.5 µg/mL like the one obtained by Chiavari-Frederico et al. [58]. For Gram-negative strains of *K. pneumoniae* isolated from urinary tract infections (UTI), the MIC concentrations were between 125 and 250 µg/mL, similar to those obtained by Gadisa and Tadesse [59].

## 4. Materials and Methods

### 4.1. Obtaining and Characterization of Tansy EOs

Tansy plants were harvested between July and August 2021 from the Sohodol Valley area, Gorj County. The plant was gathered from a soil covering, predominantly granitic, bordered to the southwest by a narrow, deep valley traversed by the Sohodol River. This entire area stands as an open clearing amidst a vast expanse of woodland, spanning approximately 10 hectares. The central reference coordinates are 45°11′13.7″ N 23°08′04.0″ E, with altitudes ranging between 550 and 700 m. This delineated area lies on the left slope of the Sohodol Valley. Only young inflorescences, unaffected by pests or mechanical factors, were used. The essential oil from the dried flowers of *T. vulgare* was obtained in a Neoclevenger distillery purchased from Pellet Lab, Houston, TX, USA. Extraction of essential oils via steam entrainment is the most widely used process. Volatile oils are very light at temperatures below the boiling point of water and are carried to the distillery’s upper outlet areas by steam. Their low density and insolubility in water facilitate their separation.

The air-dried plants were introduced into the distillery boiler with a capacity of 20 L. The ratio of distilled water to dry plants was 6 L to a volume of 3 kg. The total working time was about 6 h. We obtained 20 mL of essential oil, which we stored in a dark-colored bottle at a temperature of 4 °C. The chemical compounds in the essential oil of *T. vulgare* were identified using the gas chromatography method (i.e., Shimadzu GC-2010 Plus method) coupled with a mass spectrometer (GC-MS), coupled with a quartz capillary column. Helium (1.0 mL/min) was used as carrier gas. Additionally, 1 μL of the samples were injected into the GC (detector temperature, 280 °C). Qualitative analysis used electron impact ionization (ionization energy, 70 eV). Essential oils were identified using a digital library of mass spectral data (NIST 8.0) and literature comparisons of retention indices [60]. Quantitative GC-FID analysis was performed using a SHIMADZU GC-2010 Plus instrument equipped with an MS quartz capillary column under the same conditions as GC-MS, except that N_2_ was used as the carrier gas. The temperature of the FID detector was 250 °C. The relative concentration of the compounds was calculated by measuring the chromatographic peak areas without applying any correction factor. The applied software was AFT (Advanced Flow Technology), Fathom 9 version. The relative percentage values of separated compounds were calculated by recording the peaks in the FID chromatograms. Essential oils were identified by comparing their GC retention times on apolar and polar columns with those of the sample compounds and by comparing retention indices relative to commercial mass spectral libraries of the C6-C40 alkane series. Alkanes (C6-C40) were used as reference points to calculate relative retention indices [61,62,63].

### 4.2. Antibacterial Activity of T. vulgare EOs

For this study, we selected 2 *Staphylococcus aureus*, 2 *Acinetobacter baumannii*, 3 *Pseudomonas aeruginosa*, and 4 *Klebsiella pneumoniae* bacterial strains that were multidrug resistant, isolated from patients with different infections, hospitalized at the C.C. Iliescu Institute of Cardiovascular Diseases in Bucharest and previously characterized for their resistance phenotypes and genotypes. *S. aureus* ATCC 25923 and *P. aeruginosa* ATCC 27853 were used as control reference strains. The antibiotic susceptibility assay of the studied strains was performed using the standardized Kirby–Bauer method, according to CLSI, 2018 edition [64].

For the qualitative assay of the antibiotic-tansy EO synergisms, bacterial suspensions were made in sterile physiological water from 24 h of bacterial cultures at a density of 1.5 × 10^8^ CFU/mL according to the McFarland 0.5 turbidity standard. Each bacterial inoculum was inoculated on a plate with Müller–Hinton medium, using the “cloth” seeding technique. After drying the plates, antibiotic discs alone, as well as those impregnated with a volume of 10 μL Tansy EOs, were placed at equal distances. For testing the tansy EOs, sterile paper disks impregnated with a volume of 10 μL tansy EOs were used. The plates were incubated for 18–24 h at 35 °C ± 2 °C. After incubation, the diameters of growth inhibition zones were measured and expressed in mm. Increasing the diameter of the inhibition zone of the antibiotic in the presence of tansy EOs by ≥5 mm was the indicator of the synergistic effect.

We used the serial microdilution method for the semi-quantitative quantification of the synergistic effect of Tansy extract with antibiotics. A conventional method (according to EUCAST/2010) to obtain different concentrations of antimicrobial agents for MIC determination is to obtain a stock solution of 10,240 mg/L. By adding a volume of 19 mL of liquid Muller–Hinton broth to 1 mL of the stock solution, a final concentration of 512 μg/mL is obtained. In this study, the antibacterial activity was quantitatively determined using binary serial microdilutions in a liquid culture medium (Muller–Hinton nutrient broth) in 96-well microplates according to CLSI 2006 [65] and 2008 [66]. For this purpose, 10 binary serial dilutions, from 500 µg/mL to 1.953 µg/mL, were performed in a ratio of 9:1 nutrient broth with tansy EOs + distilled water. Bacterial suspensions of 1.5 × 10^8^ CFU/mL were prepared according to the McFarland 0.5 turbidity standard. The final volume per well was 200 µL, and the volume of bacterial suspension was 15 µL. Wells 11 and 12 corresponded to positive (bacterial culture) and negative (sterile culture medium) controls, respectively. The plates were incubated overnight at 37 °C. The minimum inhibitory concentration (MIC) was determined macroscopically by establishing the final concentration at which no increase in microbial growth and environmental turbidity was observed and via the spectrophotometric reading of the absorbance at 620 nm.

## 5. Conclusions

Our results have shown that the bioactive compounds from Tansy EOs are a rich source for developing new pharmaceuticals useful in the prophylaxis and treatment of bacterial infections produced by MDR Gram-negative and Gram-positive bacteria, as such or in combination with commonly used antibiotics. The antimicrobial action of Tansy Eos is justified by its complex chemical composition, the secondary metabolites potentially responsible for this activity being monoterpenes (Sabinene, Eucalyptol, *trans*-β-Ocimene, Linalool, Filifolone, Thujone, Camphor, *cis*-Carveol, Sabinol isovalerate), terpenoids (Chrysanthenone, Terpinen-4-ol), sesquiterpenes (Germacrenes D, Cedrol, Cubenol, ledol, Globulol, Artemisia ketone, Spirojatamol), and *trans*-Carvyl acetate. Moreover, Tansy essential oil showed MIC values ranging between 500 and 62.5 μg/mL against the tested strains, displaying synergistic activity with different classes of antibiotics. In conclusion, the presented results demonstrate that Tansy EOs hold promise for developing new antimicrobial formulations for treating active MDR bacterial infections, being a natural source of valuable bioactive compounds.

## Figures and Tables

**Table 1:** Chemical compounds of essential oil from *T. vulgare*, listed in order of elution, as identified via GC-MS analysis.

Nr. Peak	Compound	Retention Time (min)	Compound Class	Concentration (*w*/*w* %)
1	α-Thujone	4.24	MO	0.05 ± 0.02
2	α-Pinenene	4.42	MH	0.85 ± 0.03
3	Camphene	4.76	MH	0.53 ± 0.02
4	Dehydro sabinene ketone	4.83	MO	0.02 ± 0.01
5	Sabinene	5.22	MH	1.51 ± 0.01
6	β-Pinene	5.36	MH	0.42 ± 0.04
7	β-Mircene	5.54	MH	0.14 ± 0.03
8	2,3-Dehydro-1,8-cineole	5.61	MO	0.04 ± 0.01
9	α-Phellandrene	6.00	MH	0.03 ± 0.01
10	(+)-3-Carene	6.08	MH	0.16 ± 0.02
11	α-Terpinene	6.27	MH	0.29 ± 0.03
12	p-Cymene	6.46	MO	0.52 ± 0.01
13	D-Limonene	6.59	MH	0.62 ± 0.02
14	Eucalyptol	6.67	MO	2.47 ± 0.03
15	*Trans*-β-Ocimene	7.01	MH	0.07 ± 0.01
16	γ-Terpinene	7.37	MH	0.58 ± 0.01
17	*cis*-Sabinene hydrate	7.71	MO	0.02 ± 0.01
18	Terpinolene	8.16	MH	0.16 ± 0.02
19	Linalool	8.54	MO	0.03 ± 0.01
20	Filifolone	8.57	MO	0.33 ± 0.01
21	α-Thujone	8.76	MH	3.47 ± 0.03
22	β-Thujone	9.15	MO	30.26 ± 0.04
23	Chrysanthenone	9.22	MO	1.60 ± 0.01
24	*trans*-(2-Ethylcyclopentyl)methanol	9.32	O	0.09 ± 0.01
25	(+)-Isothujol	9.71	MO	0.19 ± 0.02
26	(−)-*cis*-Sabinol	9.78	MO	0.20 ± 0.03
27	L-*trans*-Pinocarveol	9.83	MO	0.19 ± 0.01
28	(+)-Camphor	10.00	MO	1.67 ± 0.03
29	Pinocarvone	10.48	MO	0.34 ± 0.01
30	(1S)-endo)-(−)-Borneol	10.79	MO	0.24 ± 0.01
31	*cis*-Pinocamphone	10.91	MO	0.03 ± 0.01
32	(−)-Terpinen-4-ol	11.06	MO	1.01 ± 0.02
34	α-Thujenol	11.12	MO	0.02 ± 0.01
35	1,5-Menthadien-7-ol	11.42	MO	0.05 ± 0.01
36	*cis*-Dihydrocarvone	11.52	MO	0.38 ± 0.03
37	*cis*-Dihydrocarvone	11.58	MO	0.12 ± 0.02
38	(−)-*trans*-Isopiperilenol	11.69	MO	0.02 ± 0.01
39	*trans*-Dihydrocarvone	11.80	MO	0.02 ± 0.01
40	*cis*-p-Menth-2-en-7-ol	11.90	MO	0.23 ± 0.01
41	*trans*-p-Menth-2-en-7-ol	11.95	MO	0.16 ± 0.02
42	*cis*-Carveol	12.28	MO	1.23 ± 0.02
43	*trans*-Chrysanthenyl acetate	12.68	MO	0.06 ± 0.01
44	Carveol	12.70	MO	0.79 ± 0.01
45	(−)-Carvone	13.05	MO	0.4 ± 0.02
46	Piperitone	13.24	MO	0.04 ± 0.01
47	*cis*-Chrysanthenyl acetate	13.48	MO	1.25 ± 0.03
48	*iso*-3-Thujyl acetate	13.61	MO	0.06 ± 0.01
49	(Z)-Linalool oxide acetat pvr	14.23	MO	0.02 ± 0.01
50	(−)-Bornyl acetate	14.36	MO	0.09 ± 0.01
51	*trans*-Sabinil acetate	14.49	MO	4.6 ± 0.03
52	*trans*-2,*trans*-4-Decadiene|C10H18	15.03	MH	0.25 ± 0.02
53	Chrysanthenyl propionate	15.59	MO	0.02 ± 0.01
54	1,4-p-Menthadien-7-ol	15.81	MO	0.07 ± 0.01
55	*cis*-Carvyl acetate	15.90	MO	0.23 ± 0.01
56	α-Fenchene	16.02	MH	0.52 ± 0.02
57	Pseudolimonen	16.49	MH	0.13 ± 0.01
58	*trans*-Carveyl acetate	16.78	MO	34.44 ± 0.03
59	7-epi-Silphiperfol-5-ene	17.26	SH	0.02 ± 0.01
60	α-Copaene	17.31	MH	0.06 ± 0.01
61	Caryophylene	18.66	SH	0.25 ± 0.02
62	β-Copaene	18.97	MH	0.02 ± 0.01
63	Humulene	19.75	SH	0.09 ± 0.01
64	γ-Cardinene	20.35	SH	0.05 ± 0.01
65	Germacrene D	20.53	SH	3.03 ± 0.02
66	Bicyclogermacren	20.97	SH	0.53 ± 0.01
67	β-Cadinene	21.66	SH	0.23 ± 0.02
68	*cis*-Verbenyl angelate	21.95	SO	0.05 ± 0.01
69	Bornyl acetate, (E)-methyl tiglate	22.84	MO	0.09 ± 0.01
70	Spathulenol	23.34	SO	0.09 ± 0.01
71	Caryophylene oxide	23.49	SO	0.11 ± 0.01
72	Salvial-4(14)-en-1-one	23.80	SO	0.03 ± 0.01
73	Cedrol	24.25	SO	0.14 ± 0.02
74	10-epi-juneol	24.64	SO	0.03 ± 0.01
75	Cubenol	24.81	SO	0.05 ± 0.01
76	13-Tetradecanolide	25.38	O	0.3 ± 0.01
77	α-Cardanol	25.58	SO	0.13 ± 0.01
78	Neointermedeol	25.64	SO	0.31 ± 0.02
79	Intermedeol	25.81	SO	0.32 ± 0.01
Total concentration				99.26

Abbreviations: MH = monoterpene hydrocarbons; MO = oxygenated monoterpenes; SH = sesquiterpene hydrocarbons; SO = oxygenated sesquiterpenes; O = others. Values are mean ± standard deviation of three different samples of *T. vulgare*, analyzed individually in triplicate. Retention time identification is based on comparing retention time with standard compounds; MS identification is based on comparing mass spectra. The amounts were calculated using calibrated curves with pure standard compounds.

**Table 2:** The main chemical compounds isolated and identified in *T. vulgare* essential oils compared to similar studies.

Identified Compound	%						
	1	2	3	4 (R)	4 (M)	4 (W)	4 (T)
Sabinene	1.51	2.02	0.1	2.09	3.39	2.36	0.24
Eucalyptol	2.47	n.d.	n.d.	n.d.	n.d.	n.d.	n.d.
*Trans*-β-Ocimene	0.07	n.d.	n.d.	n.d.	n.d.	n.d.	n.d.
Linalool	0.03	0.38	n.d.	0.33	0.33	0.28	0.17
Filifolone	0.33	0.15	tr	n.d.	n.d.	n.d.	n.d.
A-Thujone	3.47	0.08	0.9	n.d.	tr	0.14	n.d.
β-Thujone	30.26	3.66	66.6	n.d.	n.d.	n.d.	n.d.
Chrysanthenone	1.6	3.76	tr	n.d.	n.d.	n.d.	n.d.
(+)-Camphor	1.67	30.48	3.1	31.21	10.17	11.93	1.27
*cis*-Pinocamphone	0.03	n.d.	n.d.	n.d.	n.d.	n.d.	n.d.
(−)-Terpinen 4-ol	1.01	0.09	0.8	n.d.	n.d.	n.d.	n.d.
*cis*-Carveol	1.23	n.d.	n.d.	n.d.	n.d.	n.d.	n.d.
*cis*-Chrysanthenyl acetate	1.25	0.1	n.d.	0.07	0.13	1.91	0.11
(z)-Linalool oxide acetate (pyranoid)	0.01	n.d.	n.d.	n.d.	n.d.	n.d.	n.d.
*trans*-Sabinyl acetate	4.6	n.d.	0.1	n.d.	n.d.	n.d.	n.d.
α-Fenchene	0.52	n.d.	n.d.	n.d.	n.d.	n.d.	n.d.
*trans*-Carvyl acetate	34.44	n.d.	n.d.	n.d.	n.d.	n.d.	n.d.
β-Copaene	0.02	n.d.	tr	n.d.	n.d.	n.d.	n.d.
Germacrene D	3.03	0.09	0.7	0.46	0.42	0.7	0.38
Cedrol	0.14	n.d.	n.d.	n.d.	n.d.	n.d.	n.d.
Juneol	0.03	n.d.	n.d.	n.d.	n.d.	n.d.	n.d.

Abbreviations: tr—Trace amounts (<0.05%); n.d.—Not determined; 1—compounds identified in this study; 2—compounds identified by Coté et al. [24]; 3—compounds identified by Radulović et al. [27]; 4—compounds identified by Nurzyńska-Wierdak et al. [23] from several sites: R—reclaimed site; M—the meadow near the river; W—ruderal site; T—plants growing along the road.

**Table 3:** Growth inhibition zone diameters obtained for beta-lactam antibiotics tested in association with Tansy EO against the Gram-negative bacterial strains.

Strain	Diameter (mm)																						
	Tv	TPZ (100 + 10 µg)	TPZ + Tv	PIP (75 µg)	PIP + Tv	SAM (10 + 10 µg	SAM + Tv	CAZ (10 µg)	CAZ + Tv	ATM (30 µg)	ATM + Tv	IPM (10 µg)	IPM + Tv	MEM (10 µg)	MEM + Tv	GM (10 µg)	GM + Tv	TOB (10 µg)	TOB + Tv	LVX (5 µg)	LVX + Tv	CIP (5 µg)	CIP + Tv
***Pa*_ATC_**	21	20(S)	21(S)	30(S)	28(I)	20(S)	20(S)	17(S)	21(S)	16(I)	20(S)	20(S)	21(S)	24(S)	28(S)	20(S)	22(S)	18(S)	20(S)	24(S)	28(S)	20(S)	28(S)
***Pa*_3162_**	19	6(R)	12(S)	6(R)	14(I)	8(R)	19(S)	6(R)	17(S)	6(R)	16(S)	6(R)	12(S)	10(R)	19(S)	6(R)	18(S)	6(R)	19(S)	6(R)	19(S)	6(R)	14(I)
***Pa*_11I_**	18	6(R)	10(S)	6(R)	15(I)	10(R)	20(S)	8(R)	19(S)	6(R)	18(S)	6(R)	10(S)	8(R)	21(S)	6(R)	20(S)	6(R)	20(S)	6(R)	18(S)	6(R)	15(I)
***Ab*_3997_**	20	6(R)	17(S)	10(R)	18(S)	6(R)	20(S)	6(R)	20(S)	6(R)	19(S)	6(R)	17(S)	6(R)	19(S)	6(R)	17(S)	6(R)	20(S)	10(R)	18(S)	10(R)	18(S)
***Ab*_288_**	22	6(R)	20(S)	8(R)	20(S)	6(R)	22(S)	6(R)	14(S)	14(I)	20(S)	6(R)	20(S)	8(R)	24(S)	6(R)	20(S)	6(R)	22(S)	6(R)	20(S)	8(R)	20(S)
***Kp*_6I_**	21	6(R)	19(S)	8(R)	17(S)	6(R)	19(S)	14(R)	20(S)	6(R)	21(S)	6(R)	19(S)	6(R)	19(S)	6(R)	19(S)	6(R)	19(S)	6(R)	17(S)	8(R)	17(S)
***Kp*_14I_**	18	6(R)	17(S)	6(R)	19(S)	10(R)	18(S)	6(R)	19(S)	6(R)	19(S)	6(R)	17(S)	8(R)	18(S)	6(R)	17(S)	10(R)	18(S)	6(R)	19(S)	6(R)	19(S)
***Kp*_15I_**	22	6(R)	18(S)	8(R)	21(S)	10(R)	20(S)	6(R)	21(S)	14(I)	20(S)	6(R)	18(S)	10(R)	20(S)	6(R)	18(S)	10(R)	20(S)	10(R)	21(S)	6(R)	21(S)
***Kp*_19I_**	22	6(R)	20(S)	12(R)	18(S)	6(R)	18(S)	6(R)	17(S)	10(R)	20(S)	6(R)	20(S)	6(R)	18(S)	6(R)	20(S)	6(R)	18(S)	12(R)	18(S)	12(R)	18(S)

Abbreviations: *Pa* = *P. aeruginosa*, *Kp* = *K. pneumoniae*, *Ab* = *A. baumannii*, *Tv* = *T.vulgare*, TPZ = Piperacillin + Tazobactam, PIP = Piperacillin, SAM = Ampicillin + Sulbactam, CAZ = Ceftazidime, ATM = Aztreonam, IMP = Imipenem, MEM = Meropenem, GN = Gentamicin, TOB = Tobramycin, LEV = Levofloxacin, CIP = Ciprofloxacin. w/w %Tansy = 125 µg/mL; S = Susceptible; I = Intermediate; R = Resistant. Diameter of inhibition zones (mm), including disc diameter of 6 mm. Values are mean ± standard deviation of three different samples of each *T. vulgare*, analyzed individually in triplicate.

**Table 4:** The number of Gram-negative strains out of the total number of strains from each species for which a synergism with *Tv* EOs was noted.

Antibiotic Class	Antibiotics	*Pseudomonas aeruginosa*	*Acinetobacter baumannii*	*Klebsiella pneumoniae*
Penicilins/+clavulanic acid or sulbactam	TPZ	2/3	2/2	4/4
	PIP	3/3	2/2	4/4
	SAM	2/3	2/2	4/4
Cephalosporins	CAZ	2/3	2/2	4/4
Monobactams	ATM	2/3	2/2	4/4
Carbapenems	IMP	1/3	2/2	4/4
	MEM	3/3	2/2	4/4
Aminoglycosides	GN	1/3	2/2	4/4
	TOB	3/3	2/2	4/4
Quinolones	LEV	3/3	2/2	4/4
	CIP	2/3	2/2	4/4

Abbreviations: TPZ = Piperacillin + Tazobactam, PIP = Piperacillin, SAM = Ampicillin + Sulbactam, CAZ = Ceftazidime, ATM = Aztreonam, IMP = Imipenem, MEM = Meropenem, GN = Gentamicin, TOB = Tobramycin, LEV = Levofloxacin, CIP = Ciprofloxacin.

**Table 5:** Influence of Tansy extract on the antibiotic resistance profile of Gram-positive and Gram-negative strains.

Strain	The Influence of EO Tansy against Bacterial Growth in Combination with the Antibiotic (µg/mL)											
	CIP (5 µg)	CIP + *Tv*	FEP (30 µg)	FEP + *Tv*	AMC (30 µg)	AMC + *Tv*	GM (10 µg)	GM + *Tv*	IPM (10 µg)	IPM + *Tv*	TOB (10 µg)	TOB + *Tv*
*S. aureus*_732_	128	16	256	32	128	32	128	16	128	16	256	16
*S. aureus*_735_	256	32	512	16	64	16	256	32	256	32	512	16
*A. baumannii*_288_	64	8	128	8	64	8	64	16	128	64	128	32
*A. baumannii*_3997_	256	32	128	32	32	64	256	32	256	32	64	64
*P. aeruginosa*_3162_	64	16	256	8	128	32	128	8	64	16	128	16
*P. aeruginosa*_11I_	128	64	64	32	256	64	128	16	128	32	256	8
*K. pneumoniae*_6I_	256	64	256	8	256	32	16	4	512	32	128	32
*K. pneumoniae*_14I_	128	32	128	32	128	8	128	8	256	16	256	64
*K. pneumoniae*_15I_	256	64	64	16	64	32	64	16	128	8	64	8
*K. pneumoniae*_19I_	128	32	64	8	64	16	256	8	128	16	128	8

Abbreviations: FEP = Cefepime; AMC = Amoxicillin + Clavulanic Acid; TOB = Tobramycin; *w*/*w* % Tansy = 125 µg/mL.

## Data Availability

Data are contained within the article.
